# RhoT1 and Smad4 Are Correlated with Lymph Node Metastasis and Overall Survival in Pancreatic Cancer

**DOI:** 10.1371/journal.pone.0042234

**Published:** 2012-07-31

**Authors:** Hua Jiang, Chengzhi He, Shasha Geng, Haihui Sheng, Xiaoying Shen, Xiaoyan Zhang, Hang Li, Shizhang Zhu, Ximei Chen, Changqing Yang, HengJun Gao

**Affiliations:** 1 Department of Gastroenterology, Shanghai East Hospital, Tongji University School of Medicine, Shanghai, China; 2 Department of Gastroenterology, Institute of Digestive Diseases, Tongji Hospital, Tongji University School of Medicine, Shanghai, China; 3 National Engineering Center for Biochip at Shanghai, Shanghai, China; Wayne State University School of Medicine, United States of America

## Abstract

Cancer cell invasion and metastasis are the most important adverse prognostic factors for pancreatic cancer. Identification of biomarkers associated with outcome of pancreatic cancer may provide new approaches and targets for anticancer therapy. The aim of this study is to examine the relationship between the expression of RhoT1, Smad4 and p16 and metastasis and survival in patients with pancreatic cancer. The analysis showed that the high cytoplasmic expression levels of RhoT1, Smad4 and p16 in pancreatic cancer tissues had significantly negative correlation with lymph node metastasis (LNM) (*P* = 0.017, *P* = 0.032, *P* = 0.042, respectively). However, no significant association was observed between perineural invasion (PNI) and the expression of above three proteins (all *P*>0.05). Additionally, the survival analysis showed that the low expression levels of RhoT1 and Smad4 were significantly associated with worse survival (*P* = 0.034, *P* = 0.047, respectively). In conclusion, these results indicated that the low-expression levels of RhoT1 and Smad4 were significantly associated with LNM and shorter survival. RhoT1 may be considered as a potential novel marker for predicting the outcome in patients with pancreatic cancer.

## Introduction

Pancreatic ductal adenocarcinoma (PDAC) is the predominant form of pancreatic cancer, which ranks fourth in cancer-related causes of death [1]. It was estimated that 43140 Americans were diagnosed and 36800 patients died of pancreatic cancer in the United States in 2010 [2]. Despite extensive clinical and scientific efforts, the prognosis of this exceptionally lethal disease has not improved significantly over the past decades [2], [3]. The median survival after diagnosis is 3–6 months without treatment, and resectional surgery and adjuvant treatment increase median survival to around 23 months [4].

Numerous studies have found that RAS regulates the growth and metastasis of pancreatic cancer cells. The Rho family of GTPases is a subfamily of the Ras superfamily. Rho GTPases have been reported to contribute to most steps of cancer initiation and progression including the acquisition of unlimited proliferation potential, survival and evasion from apoptosis, tissue invasion and the establishment of metastases [5], [6], [7], [8], [9], [10], [11], [12]. The Rho family of GTPases contains 20 members. Most of what we know about the role of Rho GTPases in cancer cell invasion comes from the studies of the prototypic members RhoA, RhoB and RhoC, Rac1 and Cdc42. However, little is known about roles of other less-characterized family members in cancer. Some previous studies have revealed that RhoA and RhoC expression are frequently increased in human cancers, while RhoB is often down-regulated. RhoT1 belongs to the mitochondrial Rho GTPase family [13]. However, RhoT1 protein is classified as atypical GTPases because it is not regulated as the other classical GTPases [14], [15]. So far little is known about the role of RhoT1 in cancer progression. In a previous study, we have identified a total of 1276 genes that are differentially expressed in PDAC. Among them, 691 genes are up-regulated and 585 genes are down-regulated genes, including RhoT1, Smad4 and p16 [16]. Therefore, we are interested in knowing whether the RhoT1 is similarly involved in the development of cancer.

In addition, previous studies have reported that mutation of Smad4 is identified in approximately 50% of pancreatic adenocarcinomas [17]. A number of studies have shown that loss of Smad4 is generally observed in pancreatic carcinogenesis, and inactivation of Smad4 is associated with poor prognosis in pancreatic cancer [18], [19]. Similarly, it has been demonstrated that loss of p16 expression is observed in most pancreatic tumor [20], [21], and constitutes a key event in the multistep process of pancreatic ductal cell transformation. However, the significance of p16 and Smad4 inactivation for complex and tissue-specific aspects of pancreatic cancer progression, such as angiogenesis and metastasis, is less understood [22]. Also, there are relatively few studies that have examined the possible role of Smad4 and p16 in the progression of LNM and PNI in pancreatic cancer.

Cancer cell invasion and metastasis are critical steps in pancreatic cancer progression, and are the main causes of poor survival in pancreatic cancer. Predicting invasion and metastasis for patients with pancreatic cancer may provide important insights into the pancreatic cancer progression and prognosis. Identification of biomarkers associated with outcome of pancreatic cancer can provide new approaches and targets for anticancer therapy. Therefore, we have carried out this study to examine the expression of RhoT1, Smad4 and p16 in pancreatic cancer, and to analyze whether the expression patterns of RhoT1, Smad4 and p16 are correlated with metastatic potential and are predictive of clinical outcome in patients with pancreatic cancer.

## Results

### Patient Characteristics

As shown in Table 1, the sample consisted of 162 patients with a diagnosis of pancreatic cancer (102 men and 60 women). The mean age at diagnosis was 59 year-old (range 34 to 85 year-old). The median tumor size was 4 cm (range 0.5 to 14 cm). Most tumors (117/162, 72%) were well differentiated, 8 (8%) were moderately differentiated, and 31 (19%) were poorly differentiated. 80 (49.4%) tumors were categorized as American Joint Committee on Cancer (AJCC) stage I, 80 as (49.4%) stage II, none as stage III, and 2 (1.2%) as stage IV. 83 (51.2%) of 162 patients had PNI. 65 (40.1%) of 162 patients had LNM, The median number of lymph nodes (LNs) examined was 7. The median number of LNs assessed in the node-positive patients was 10 compared with a median of 6 lymph nodes in the node-negative patients (*P* = 0.007).

**Table 1:** Clinicopathological characteristics of the pancreatic cancer.

Characteristics	No. of Patients	%
Age (years)		
median	59	
range	34–85	
Gender		
female	60	37
Male	102	63
Stage		
stage I	80	49.4
stage II	80	49.4
stage III	0	0
stage IV	2	1.2
Lymph node status		
negative	97	59.9
positive	65	40.1
Lymph node ratio (LNR)		
LNR = 0	97	59.9
LNR>0, <0.5	50	30.9
LNR≧0.5	15	9.2
Perineural invasion		
negative	79	48.8
positive	83	51.2
Tumor size		
median (cm)	4	
range (cm)	0.5–14	
Tumor differentiation		
well	117	72.2
moderate	14	8.7
poor	31	19.1

### Associations between the Various Clinicopathological Factors and the Presence of LNM and PNI

Associations between the various clinicopathological factors and the presence of LNM and PNI were analyzed to identify the risk factors of LNM and PNI (Table 2). These factors included: gender, age (<60 years or ≧60 years), location of tumor (head or body and rear), long diameter of tumor (<4.0 cm or ≧4.0 cm), tumor differentiation (poor or moderate/well). Location of tumor was significantly associated with LNM (*P* = 0.03). There was no significant association between LNM and age, gender, long diameter of tumor and tumor differentiation (*P*>0.05 for each). In addition, no significant association was observed between PNI and age, gender, location of tumor, long diameter of tumor and tumor differentiation (all *P*>0.05).

**Table 2:** Associations between the various clinicopathological factors and the presence of lymph node metastasis and perineural invasion.

Variables	lymph node metastasis			perineural invasion		
	Positive	Negative	*P* value	Positive	Negative	*P* value
	*n = *65	*n = *97		*n = *83	*n = *79	
Age (years)						
<60	39	50	0.491	41	48	0.146
≧60	26	47		42	31	
Gender						
female	22	38	0.289	28	32	0.372
male	43	59		55	47	
Location						
head	53	64	0.03	59	58	0.74
body/rear	12	33		24	21	
Size						
<4	33	41	0.287	38	36	0.978
≧4	32	56		45	43	
Differentiation						
poor	11	20	0.558	17	14	0.655
moderate/well	54	77		66	65	

### Expression of RhoT1, Smad4 and P16 in Pancreatic Cancer Tissues and Paracancerous Tissues

**Figure 1:**
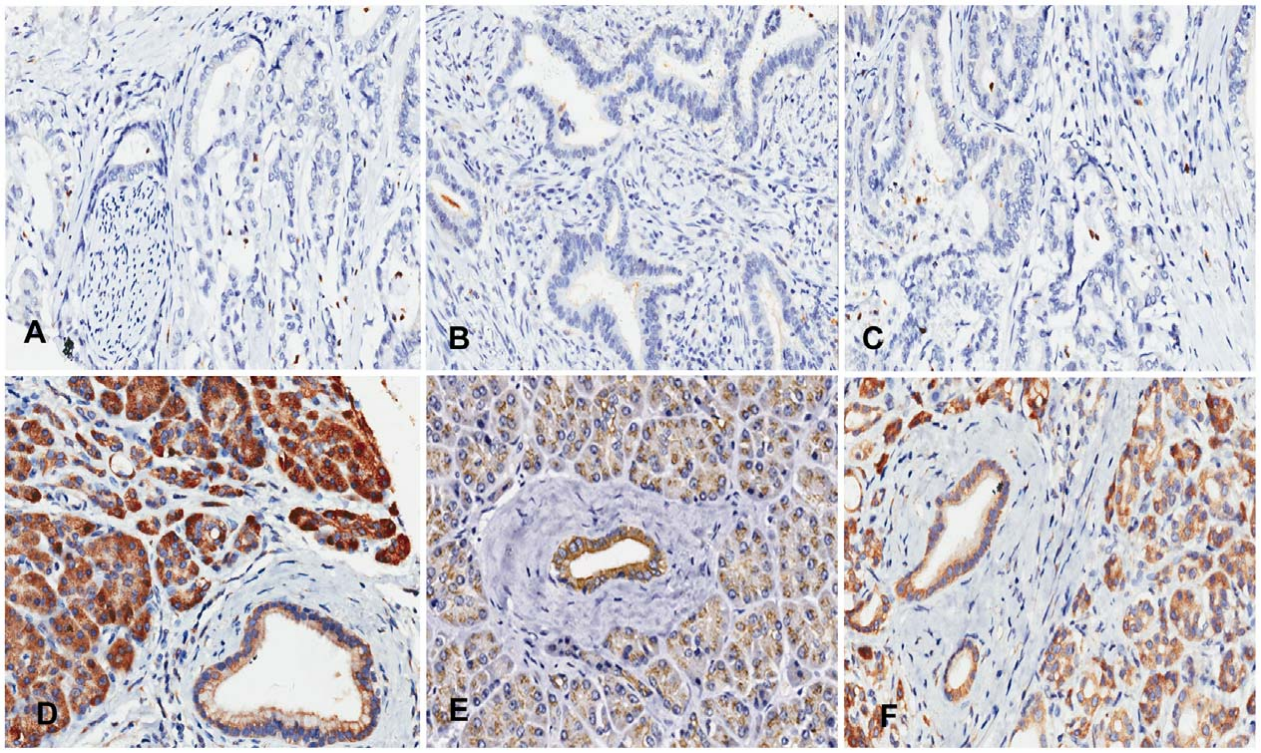
Panels A, B and C respectively show the negative expression of RhoT1, Smad4 and p16 in the cancer tissues. Panels D, E and F respectively show the positive expression of RhoT1, Smad4 and p16 in the paracancerous tissues. All images were taken at 200× magnification.

RhoT1 and p16 staining were localized to the cytoplasm (Figure 1). Although, Smad4 was expressed mainly in the cytoplasm (Figure 1), some Smad4 was seen in the nucleus. The cytoplasmic expression levels of RhoT1, Smad4 and p16 were lower in cancer tissues than paracancerous tissues (*P*<0.0001, *P*<0.0001, *P* = 0.002, respectively). There was a strong correlation between cytoplasmic and nuclear Smad4 expression (Spearman correlation coefficient  = 0.321; *P*<0.0001). However, no significant difference was observed in nuclear Smad4 expression between cancer tissues and paracancerous tissues (*P*>0.05).

### Associations between the Expression Levels of RhoT1, Smad4 and P16 and the Clinicopathological Features

The analysis showed that high cytoplasmic expression level of RhoT1 was significantly negative correlation with LNM in cancer tissues (*P* = 0.003). The difference remained significant even after adjustment for age, gender, cancer location and long diameter of tumor (*P* = 0.017). Similarly, cytoplasmic expression levels of Smad4 and p16 in cancer tissues were also negatively correlated with LNM (*P* = 0.032, *P* = 0.042, respectively). However, there were no significant associations between PNI and the expression levels of three proteins (all *P*>0.05; Table 3). Since no significant difference was observed between cancer tissues and paracancerous tissues, nuclear expression of Smad4 was eliminated from further testing the association with the clinicopathological features. Logistic regression analysis showed that the cytoplasmic expression level of RhoT1 in cancer tissues acted as an independent risk factor for LNM (*P = *0.042). However, according to the analysis, the cytoplasmic expression levels of Smad4 and p16 could not be used as independent risk factors for LNM (*P*>0.05). Additionally, the results showed that no significant association was observed between the cytoplasmic expression levels of RhoT1, Smad4, p16 and clinicopathological features, which including age, gender, tumor location, size, tumor differentiation, and AJCC stage (all *P*>0.05; Table 4).

**Table 3:** Associations between the cytoplasmic expression of RhoT1, Smad4, p16 and the presence of lymph node metastasis and perineural invasion.

Variables	lymph node metastasis			perineural invasion		
	Positive	Negative	*P* value	Positive	Negative	*P* value
	*n = *65	*n = *97		*n = *83	*n = *79	
RhoT1 expression						
high	12	43	0.003	23	32	0.198
low	53	54		60	47	
Smad4 expression						
high	8	35	0.032	25	18	0.304
low	57	62		58	61	
p16 expression						
high	7	29	0.042	20	16	0.35
Low	58	68		63	63	

**Table 4:** Associations between the cytoplasmic expression of RhoT1, Smad4, p16 and the clinicopathological features

Variables	RhoT1 expression			Smad4 expression			p16 expression		
	low	high	*P* value	low	high	*P* value	low	high	*P* value
Age (years)									
<60	60	29	0.922	70	19	0.097	70	19	0.299
≧60	47	26		49	24		56	36	
Gender									
female	36	24	0.660	38	22	0.382	48	12	0.897
male	71	31		81	21		78	24	
Location									
head	80	37	0.876	88	29	0.744	94	23	0.468
body/rear	27	18		31	14		32	13	
Size									
<4	47	27	0.324	52	22	0.186	59	15	0.114
≧4	60	28		67	21		57	21	
Differentiation									
moderate/well	81	45	0.278	94	37	0.808	102	29	0.149
poor	21	10		25	6		24	7	
AJCC stage									
stage I	46	34	0.191	50	30	0.242	56	24	0.33
stage II	60	20		68	12		69	11	
stage IV	1	1		1	1		1	1	

**Figure 2:**
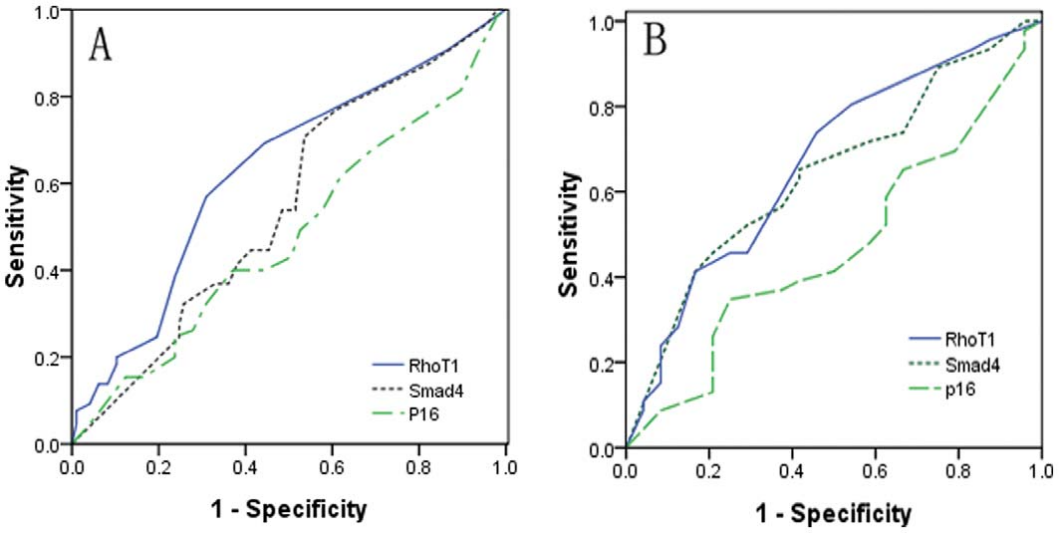
A shows the Receiver operator characteristic (ROC) curve analysis using the expression of RhoT1, Smad4 and p16 for discriminating LNM (*n* = 162). The area under the ROC curve (AUC) for RhoT1, Smad4 and p16 were 0.629, 0.551, and 0.480 respectively. B Shows the ROC curve analysis of the expression of RhoT1, Smad4 and p16 for overall survival (*n = *70). The AUC values for three proteins were 0.667, 0.643, and 0.469, respectively.

Additionally, the ROC curves were established to assess the potential diagnostic value of these proteins for predicting between specimens with LNM and specimens without LNM. The analysis discovered that the area under the ROC curve (AUC) for RhoT1 was 0.629 [95% *confidence interval* (*CI*) 0.541 to 0.718], the AUC for Smad4 was 0.551 (95% *CI* 0461 to 0.640) and the AUC for p16 was 0.480 (95% confidence interval 0.388 to 0.572). The statistical analysis indicated that only AUC value for RhoT1 was significantly greater than 0.5 (*P = *0.005). At the cutoff of 75% (relative expression positive-rate), sensitivity of the RhoT1 was 69.2% and specificity was 55.7% (Figure 2–A).

### Survival Analysis

The survival analysis showed that the survival rates after 5 years was 28.6%, median survival was 14 months. The analysis of survival showed LNM, tumor differentiation and stage (I *v* II) as being negatively significant predictors of pancreatic cancer OS (*P = *0.01, *P = *0.003, *P = *0.036, respectively), and patients with an Lymph node ratio (LNR) >0 to <50% had a longer median survival (11 months) compared with patients who had an LNR ≧50% (8 months) (*P* = 0.035) (data not shown). In contrast, the evaluation of OS by age, group, gender, tumor location, tumor size, and PNI did not indicate any significant differences (*P*>0.05 for all). Two patients with stage IV were excluded from the survival analysis because the sample size was too small.

**Figure 3:**
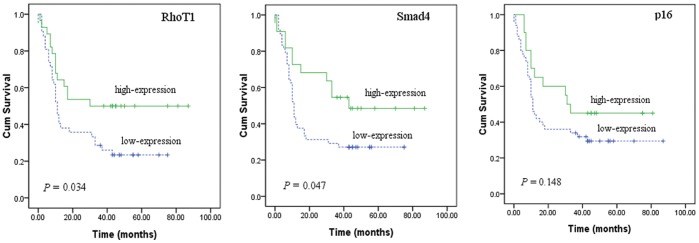
Kaplan–Meier curves showing correlation between high or low expression of RhoT1, Smad4, p16 and overall survival in patients with pancreatic cancer (*n = *70). High expression levels of RhoT1 and Smad4 correlated with better survival (*P* = 0.034, *P = *0.047, respectively; log rank test), no significant association was observed between the expression level of p16 and overall survival (*P*>0.05).

In addition, the 5-year survival rates were 23.4%, 27.1% and 50%, 48.5% in patients with low-expression and high-expression of RhoT1 and Smad4 respectively. Patients with high cytoplasmic expression levels of RhoT1 and Smad4 achieved better survival than patients with low-expression levels of RhoT1 and Smad4 (*P = *0.034, *P = *0.047, respectively; Figure 3). However, there was no statistically significant relationship in OS between patients with high or low expression of p16 (*P = *0.148). The AUC values for RhoT1, Smad4 and p16 were 0.667 (95% *CI* 0.532 to 0.802), 0.643 (95% *CI* 0.509 to 0.777), and 0.469 (95% *CI* 0.328 to 0.610) respectively. Additionally, the analysis showed that AUC value for RhoT1 was significantly greater than 0.5 (*P = *0.022). However, no significant difference was observed between areas of Smad4 and p16 in ROC analysis (*P = *0.051, *P = *0.674, respectively) (Figure 2–B).

Table 5 shows the results of univariate Cox proportional hazards analysis for the major clinicopathologic features and for the cytoplasmic expression of RhoT1, Smad4 and p16 in pancreatic cancer tissues. The analysis showed that the variables with an increase in the risk of death included poor differentiation [*hazard ratio* (*HR*), 2.484; 95% *CI*, 1.329–4.642; *P = *0.004], Higher stage (*HR*, 1.82; 95% *CI*, 1.01–3.28; *P = *0.044) and LNM (*HR*, 2.09; 95% *CI*, 1.16–3.77; *P = *0.014) and the low-expression of RhoT1 (*HR*, 1.92; 95% *CI*, 1.02–3.61; *P = *0.042). Multivariate analysis by Cox regression and correction for all histopathologic features and RhoT1 expression revealed that tumor differentiation was an independent prognostic factor (*P = *0.01), whereas tumor stage, LNM and RhoT1 expression were not statistically significant (*P = *0.623, *P = *0.101, *P = *0.3, respectively) (Table 5).

**Table 5:** Univariate and multivariable Cox regression analysis of overall survival (*n = *70).

Features	Univariate analysis		Multivariable analysis	
	*HR* (95% *CI*)	*P* value	*HR* (95% *CI*)	*P* value
Tumor features				
age (years),<60 *v* ≧ 60	1.38(0.77 to 2.46)	0.273		
sex, female *v* male	0.60(0.32 to 1.13)	0.114		
tumor location, head *v* body/rear	1.08(0.59 to 1.99)	0.787		
tumor size, <4 *v* ≧ 4	1.41(0.79 to 2.52)	0.239		
tumor differentiation, poor *v* moderate/well	2.48(1.32 to 4.64)	0.004	2.49(1.24 to 5.01)	0.010
AJCC stage, II *v* I	1.82(1.01 to 3.28)	0.044	1.29(0.46 to 3.66)	0.623
LNM, positive *v* negative	2.09(1.16 to 3.77)	0.014	2.35(0.84 to 6.57)	0.101
PNI, positive *v* negative	1.57(0.88 to 2.82)	0.124		
Expression of proteins, low *v* high				
RhoT1	1.92(1.02 to 3.61)	0.042	1.43(0.72 to 2.84)	0.300
Smad4	1.94(0.98 to 3.84)	0.056		
p16	1.62(0.82 to 3.20)	0.162		

Note: *HR*: Hazard ratio; LNM: lymph node metastasis; PNI: perineural Invasion.

## Discussion

Current studies have reported that molecular markers can provide crucial information for understanding pancreatic cancer progression better. Comparing the protein expression of cancer specimens with different invasion and metastasis status may provide a clue for novel metastasis-associated factors. In the present study, we found that there were significant differences of the cytoplasmic expression of RhoT1, Smad4 and p16 between cancer and paracancerous tissues. These results implied that RhoT1, Smad4 and p16 were involved in the progression of pancreatic cancer. Furthermore, low cytoplasmic expressions of RhoT1 and Smad4 were associated with LNM and worse survival in patients with pancreatic cancer.

RhoT1 belongs to the mitochondrial Rho GTPase family and is first reported by Fransson *et al* in 2003 [23]. Previous studies showed that Rho-GTPases were involved in the regulation of a wide variety of cellular processes, and played important roles in carcinogenesis, cancer cell migration, invasion and metastasis. For example, the expression of RhoA and RhoC was often increased in human tumors, over-expression of RhoA and RhoC in tumors were associated with metastasis and invasion [24]. In contrast, RhoB was often down-regulated, low-expression of RhoB was inversely correlated with tumor aggressiveness [25], [26], [27], [28]. These results indicated that there were differences in the expression patterns of Rho family members in human cancer. In this study, we found that the cytoplasmic expression level of RhoT1 in cancer tissues was lower than that in paracancerous tissues, and was significantly decreased in patients with LNM compared with those without LNM (*P = *0.017) after adjustment for age, gender, cancer location and long diameter of tumor. Like RhoB, high-expression of RhoT1 was negatively correlated with tumor aggressiveness. However, its exact molecular mechanism is largely unknown. Several previous studied have demonstrated that RhoT1 is implicated in regulation of mitochondrial homeostasis and apoptosis [15], [23], a possible mechanism might be that cancer cell is resistant to RhoT1-mediated apoptosis and allows to avoid apoptotic cell death, which results in the initiation and progression of cancer. To our knowledge, this is the first study demonstrating that the cytoplasmic expression of RhoT1 is involved in the progression of pancreatic cancer. Collectively, these results suggest that RhoT1 may be a novel tumor suppressor gene of pancreatic cancer, and the low cytoplasmic expression of RhoT1 may serve as a potential predictor for the tendency to metastasize. However, no significant association between RhoT1 and PNI was found in present study. This indicates that the differences in mechanisms may exist between LNM and PNI. Further studies are clearly needed to elucidate the mechanism of RhoT1 involved in pancreatic cancer.

Additionally, Smad4 is one of the most commonly inactivated genes in pancreatic cancer, and loss of p16 expression is observed in most pancreatic cancer [20], [21]. In this study, we found that the low expression of Smad4 was associated with LNM, which was consistent with the results of some previous studies [29], [30], [31]. Tanaka *et al* [32] reported that Loss of Smad4 protein expression and chromosome 18q deletion were distinctly associated with metastasis. In another study, Tanaka *et al* also found that the expression of Smad4 was weaker in the lymph node positive group compared with the negative group (*P = *0.00075) [33]. The results indicated that Smad4 inactivation was an essential molecular event in the process of LNM. Moreover, we found that low expression of p16 was also correlated with LNM, which was consistent with following two studies. Zhi-jie Fu *et al* [34] reported that p16 expression in laryngeal squamous cell carcinoma (LSCC) was down-regulated with cervical LNM (*P*<0.05), the down-expression of p16 may be an important predictor for cervical LNM in patients of LSCC. Daniela *et al* [35] suggested that positive cytoplasmic p16 staining may be related to a lower metastatic potential of primary malignant melanoma. Thus, our study further confirmed that Smad4 and p16 played important role in the process of LNM in pancreatic cancer.

In present study, we further examined the correlation between the cytoplasmic expression levels of RhoT1, Smad4 and p16 and OS. We found that patients whose cancers had low cytoplasmic expression level of Smad4 had significantly worse survival than patients with high cytoplasmic expression of Smad4 (*P = *0.047). This finding was consistent with some previous research [36], [37]. In addition, the possible correlation between the expression of some members of Rho GTPases and clinical outcome has previously been described. For example, Takao *et al* [38]suggested that Rac1 was involved in LNM of urothelial carcinoma of the upper urinary tract, and associated with a shorter disease-free survival time (*P*<0.01) and shorter OS (*P*<0.001). Similarly, another study [28] reported that the high-expression of RhoA and RhoC were associated not only with muscle invasion and LNM (*P*<0.001, *P*<0.05, respectively), but also with poorer survival in bladder cancer (*P*<0.0001). Inversely, high-expression of RhoB was correlated with better overall survival (*P*<0.05). However, so far few studies have investigated the relationship between the expression of RhoT1 and the outcome or prognosis for patients with pancreatic cancer. In this study, we found that high RhoT1 expression was inversely correlated with survival of patients with pancreatic cancer (*P = *0.034). Moreover, the results of univariate Cox proportional hazards analysis for the cytoplasmic expression of RhoT1, Smad4 and p16 in pancreatic cancer tissues showed that the low-expression of RhoT1 was correlated with an increase in the risk of death (*P = *0.042). Taken together, the present study suggests that RhoT1 may be considered as a potential prognostic biomarker for overall survival, and as a potential therapeutic target for intervention in patients with pancreatic cancer.

In conclusion, our findings highlight that low cytoplasmic expression levels of RhoT1 and Smad4 were significantly associated with increasing risk of LNM and poorer survival. To the best of our knowledge, this is the first report demonstrating that the expression of RhoT1 may potentially be used to predict the outcome of patients with pancreatic cancer. More studies with a larger number of cases are necessary to validate our findings.

## Materials and Methods

### Patient Population

The study was approved by the ethical committee of Biobank Center related hospitals. Samples with informed consent were collected between 1995 and 2009 from 162 patients who underwent pancreatic surgery, and were stored at Biobank Center of National Engineering Center for Biochip at Shanghai. 162 patients with both clinical data and adequate tissue for inclusion in this study were identified. Clinical information, included age, gender, presentation and pathologic findings included tumor size, stage, differentiation, perineural invasion and lymph node status, were obtained from original pathology reports. Pathologic staging was updated according to current American Joint Committee on Cancer guidelines. Since we collected and analyzed the data retrospectively, follow-up data were not available in all cases. With a cut-off date of December 2011, 92 patients in our study were lost to follow-up, 70 patients with pancreatic cancer were included in our final survival analysis. Overall survival was measured from time of definitive operation to death from pancreatic cancer. Until now, 46 of the 70 patients died.

### Tissue Microarray Construction

Original formalin-fixed, paraffin-embedded specimens were used to construct a PDAC tissue microarray (FFPE TMA). Hematoxylin and Eosin (H&E)-stained standard slides from each tumor specimen were reviewed by a single pathologist (MR), who was blinded to specimen protein expression status. Representative tumor regions and its paracancerous nonmalignant pancreatic specimens (NMPs) were selected from each tissue block and 2 tissue cores (0.6 mm in diameter) were taken from each region using an automated tissue arrayer (Beecher Instruments, Sun Prarie, WI). Cores were transferred to individual recipient blocks. In all cases, cores were taken normal adjacent pancreas were also used as internal controls. Five-micron sections were cut from each recipient block. Sections were stained with H&E to confirm the presence of tumor within each core.

### Immunohistochemistry and Scoring

This is the first manuscript based on our pancreatic TMA. TMA slides were deparaffinized, rehydrated through graded alcohol, washed with Tris-buffered saline, and processed using a streptavidin– biotin–peroxidase complex method. Antigen retrieval was performed by microwaveheating sections in 10 mm sodium citrate buffer (pH 6) for 10 minutes. After quenching of endogenous peroxidase activity and blocking of nonspecific binding, 3 antibodies (RhoT1, Santa company, polyclone antibody, expression in cytoplasm; Smad4, Abcam company, monoclone antibody, expression in cytoplasm and nucleus; p16, BD company, monoclone antibody, expression in cytoplasm) were added at a special dilution, 1∶15, 1∶15,1∶300 respectively, after which slides were incubated at 4°C overnight. The corresponding secondary biotinylated rabbit antibody was used at a special dilution for 30 minutes at 37°C. After further washing with Tris-buffered saline, sections were incubated with StrepABComplex/horseradish peroxidase (1∶100, DAKO) for 30 minutes at 37°C. Chromogenic immunolocalization was performed by exposure to 0.05% 3,3-diaminobenzidine tetrahydrochloride. Other cores containing PDAC served as positive controls for those genes expression. Normal serum was used in the place of primary antibody as a negative control. Slides were counterstained with hematoxylin before dehydration and mounting.

Immunohistochemical stains were scored semi-quantitatively according to the percentage and intensity of positive-staining epithelium cells (cytoplasmic and nuclear staining of epithelium cells were scored independently): 1, 0 points for no staining; 2, 1 point for <20%; 3, 2 points for 20–75%; 4, 3 points for >75%, as described previously [39]. Next, the average intensity of immunoreactivity was graded on a scale of 0 to 3 (0, none; 1, weak, 2, intermediate; and 3 strong). The total score was the product of the scores for the intensity and positive rate of staining (Staining index  =  intensity × positive rate; absent, 0; mild, 1–3; moderate, 4–6; and strong, 7–9). For data analysis, Staining index scored as either absent or mild were considered low-expression, either moderate or strong were considered high-expression. Slides were reviewed by 2 independent observers blinded to clinical and pathologic data. In cases of disagreement, a consensus was reached by joint review.

### Statistical Analysis

The association between individual clinicopathological variables and LNM, between those proteins expression and LNM were statistically analyzed using the χ^2^ -test. Independent risk factors for LNM were determined using logistic regression analysis to identify those variables independently associated with metastasis. The optimal sensitivity and specificity of expression levels of three proteins were evaluated by receiver operating characteristic (ROC) curve analysis. The Kaplan-Meier method was used to estimate the survival function, and the log-rank test was used to examine statistical significance. Cox proportional hazards model was conducted to estimate hazard ratios for OS and to test for independent prognostic factors. All statistics were two-tailed with *P* value <0.05 considered statistically significant. Analyses were performed using the SPSS software package (version 17.0).
